# Immunotherapy for triple negative breast cancer: the end of the beginning or the beginning of the end?

**DOI:** 10.1007/s10555-022-10060-4

**Published:** 2022-09-06

**Authors:** Marek Z. Wojtukiewicz, Magda Pogorzelska, Barbara Politynska

**Affiliations:** 1 Department of Oncology, Medical University of Bialystok, Bialystok, Poland; 2 Department of Clinical Oncology, Comprehensive Cancer Center, Bialystok, Poland; 3 Department of Psychology and Philosophy, Medical University of Bialystok, Bialystok, Poland

**Keywords:** Triple negative, Breast cancer, TNBC, Immunotherapy, Future targets

Triple-negative breast cancer (TNBC), which comprises 10–20% of all breast malignancies, remains a subtype with the most dismal prognosis. TNBC cells, due to lack of estrogen (ER), progesterone (PR), and human epidermal growth factor receptor-2 (HER-2) receptor expression, are insensitive to all known endocrine and anti-HER-2-targeted therapies. As a result, chemotherapy remains the standard of care for this subgroup of patients, in whom a high recurrence rate and poor overall survival are observed.

The biology of TNBC is distinctive, not only in comparison to other breast cancers, but its natural course differs among individuals. This heterogeneity makes it even harder to define new therapeutic targets or predictive factors. Therefore, an urgent need to develop effective therapies exists, as only 20% of TNBC patients treated in a neoadjuvant and adjuvant setting achieve a pathological complete response (pCR), which is a strong surrogate marker for longer overall survival (OS). The remaining 80% are exposed to highly toxic treatment that causes a significant decrease in quality of life, while at the same time providing little clinical benefit. The efficacy of palliative chemotherapy is even more disappointing. Following the spread of the disease, most commonly to visceral organs or to the brain, the mean survival time is only 10.2 months [1].

Initially, the success of immunotherapy in treating malignancies exhibiting a pessimistic prognosis, such as malignant melanoma or lung cancer, raised hope for TNBC patients. Despite the absence of immunological targets, TNBC can potentially respond to immune checkpoint inhibitors because of the relatively high tumor mutational burden (TMB) (> 10 mutations/Mb) and the high number of tumors infiltrating lymphocytes (TILs) compared to other types of breast cancer (BC). An elevated TMB correlated with increased response to immuno-oncological drugs in multiple clinical trials, of which five evaluated the efficacy of a combination of anti-programmed cell death-1 (PD-1)/programmed cell death ligand-1 (PD-L1) and neoadjuvant chemotherapy in TNBC cancer patients. This strategy turned out to be beneficial in randomized clinical trials, as adding immunologically active drugs to preoperative chemotherapy significantly improved pCR rates found in the post-operative material. Two trials, KEYNOTE-522 (phase III) and GeparNuevo (phase II), revealed similar results in terms of event-free survival/invasive disease-free survival (EFS/iDFS). KEYNOTE-522 was a trial that enrolled 1174 early-stage TNBC patients, who received paclitaxel-carboplatin-based neoadjuvant chemotherapy followed by doxorubicin-cyclophosphamide treatment with pembrolizumab (*n* = 784) or placebo (*n* = 390). After surgery, anti-PD-1 drugs (or placebo) were administered for up to 9 cycles. Pembrolizumab + chemotherapy resulted in significant improvement in pCR (64.8% vs 51.2%, *p*-value 0.01). Analysis of data obtained up to a cut-off of March 23, 2021, revealed a positive influence on EFS (84.5% vs 76.8%), disease-free survival (DFS; 87% vs 80.7%), and OS (89.7% vs 86.9%). These results replicated the GeparNuevo data, in which 3-year iDFS in the durvalumab group reached 84.9% vs 76.9% for placebo (*p* = 0.0559). However, the trial did not meet its primary endpoint, as the pCR rate in the immunotherapy group was clinically relevant, but not statistically significant (53.4% vs 44.2%, *p* = 0.287). A phase 3 clinical trial, IMpassion130 trial (NCT02425891), led to the accelerated approval of atezolizumab in PD-1 positive TNBC patients in March 2019, as IMpassion130 and IMpassion131 both demonstrated statistically significant benefit in progression-free survival (PFS). The accelerated approval was then withdrawn in September 2021, as results published in *Annals of Oncology* suggested a failure in meeting the study’s primary endpoint of PFS superiority in the PDL-1 positive group. In addition, no survival benefit was observed (HR 1.11, 95% CI 0.76–1.64) either in the PDL-1 ( +) or in the intention to treat (ITT) population [2, 3]. At present, only pembolizumab plus chemotherapy is regarded as a viable option for both early and advanced TNBC patients. Based on these findings, some physicians have started to doubt the utility of immunotherapy for TNBC. The question thus arises: does this herald the end of immunotherapy for TNBC or conversely is the concept correct but needs to be examined from a different perspective?

## Future perspectives

Three strategies are evolving for the immunological treatment of malignancies. The first is based on the fact that the immunological activity of T-lymphocytes can be up- or down-regulated by stimulation of co-stimulatory (CD28, OX40 also known as CD134, CD137) and co-inhibitory receptors (Cytotoxic T-lymphocyte Antigen-4 (CTLA-4), PD-1, T-cell immunoglobulin (TIM-3) domain and mucin domain 3, Lymphocyte activating gene 3 (LAG-3)). Maintaining a balance between these two groups allows for the appropriate initiation of the immune response. Malignant neoplasms may deregulate the function of immune checkpoint inhibitors and activating pathways, which leads to uncontrolled growth and invasion. Anti-CTLA-4 and anti-PD-1/PDL-1 inhibitors are now one of the mainstays of oncology, yet, as mentioned above, this approach has been only partially successful in the treatment of TNBC. Nonetheless, immunotherapy is an area being actively explored in search of new potential therapeutic approaches [2].

A second promising strategy involves enhancing the anti-tumor response of CD8 + cells. The number of CD8 + lymphocytes, which are an essential element of TILs circulating in the tumor immune microenvironment (TIME), plays a key role in the immunological response, and TNBC is characterized by one of the highest levels of CD8 + . Targeting the intracavitary stimulator of the interferon gene (STING) pathway by the poly-ADP-ribose polymerase (PARP) inhibitor, Olaparib, enhances CD8 + and natural killer (NK) mediated anti-tumor activity in (BRCA-1) deficient TNBC models. This has led to the conclusion that incorporating PARP inhibitors in chimeric antigen receptor T-cell therapy (CAR-T therapy) may help achieve a response in TNBC patients. Additionally, the STING pathway can also be targeted directly, as intratumor injection of STING ligand R inhibits the growth of breast tumors [4]. Combining STING agonists with OX40 and PDL-1 receptor modulators inhibits tumor growth and augments the immunological response in mice. The safety and efficacy of humanized agonist anti-OX40 mAb, ABBV-368, is now being evaluated in locally advanced and metastatic breast cancer (NCT03893955). Another co-stimulatory molecule, the LAG-3 receptor expressed on cytotoxic T-cells, is a potential target for successful TNBC treatment. Major histocompatibility complex II (MHC II) on antigen-presenting cells (APCs) and galectin 3 found on the surface of several forms of solid tumors, including breast cancer, are two ligands for the LAG-3 receptor. Activation of the LAG-3 receptor leads to the exhaustion of cytotoxic T-cells followed by decreased anti-tumor activity. LAG-3 also has been found on regulatory T-cells. When activated, it also inhibits the cytotoxic T-lymphocyte response [5]. Several ongoing trials are now analyzing the therapeutic value of a combination of LAG-3 stimulators and PD1/PDL-1 inhibitors (NCT04252768, NCT00349934, NCT02614833, NCT03499899, NCT03742349).

A third strategy supported by early research provides promising results for leveraging inhibition of epigenetic mechanisms by DNA methyltransferase inhibitors (DNMTis), histone deacetylase inhibitors (HDACis) and, an inhibitor of histone methylation on histone H3 at lysine 27 (EZH2is). These pathways improve the response to immune checkpoint inhibitors. For example, DNMTis have multipotential effects, activation of expression of endogenous retroviral double stranded RNAs (EVRs) being the most important. The EVRs are typically hypermethylated and do not undergo transcription; however, they activate MHC I expression and initiate an interferon I–mediated response. Tumors characterized by high EVR expression respond better to CTLA-4 (melanoma) and PD1/PDL-1 inhibitors (renal cell carcinoma). The latest research results connect increased EVR expression in various malignancies, including breast cancer, to increased activation of CD8 + cells [6].

Apart from enhancing the immune response, the possibility of recruiting new therapeutic targets is also being actively explored to identify the most successful strategy for TNBC patients. Cathepsin-D (Cath-D) is an aspartic endoprotease hyper secreted by TNBC cells and associated with poor prognosis. High Cath-D levels correlate with increased BC cell proliferation and intensified angiogenesis. Anti-Cath-D therapy increases antitumor cytokine secretion and activates NK cells while reversing the immunosuppressive microenvironment of the tumor by inhibiting recruitment of tumor associated macrophages and myeloid-derived suppressor cells. This therapy raises hope for TNBC patients, as promising results of pre-clinical research have recently been published [7].

Another recently identified therapeutic target is glycolipid-2 (GD2). *In vivo*, tumor growth is suppressed by the anti-GD2 antibody—dinutuximab. This antibody extends the survival of mice bearing TNBC tumors by directing NK cells to GD2 + breast tumors and induces antibody-dependent cellular toxicity (ADCC). Therefore, combining anti-GD2 therapy and NK adoptive transfer raises the possibility of a new treatment strategy. Tumor-associated antigen mesothelin (MSLN), overexpressed in malignancies such as TNBC or mesothelioma, has also become a target of interest. MSLN plays a critical role in activating the T-cell mediated immune response. Targeted MSLN immunotherapies are now under investigation in pancreatic and lung cancers and may be potentially successful in treating triple negative breast cancers [8].

Yet another promising strategy emerged when researchers started evaluating the immunosuppressive features of extracellular adenosine, which is a key negative regulator of the immunological response. The tumor microenvironment is hypoxic by nature, and hypoxia is one of the hallmarks of cancer, responsible for tumor progression and insensitivity to anti-cancer treatment. Hypoxia intensifies adenosine triphosphate (ATP) conversion to adenosine diphosphate (ADP) and adenosine monophosphate (AMP), which is then metabolized to adenosine by CD73 found on the surface of tumor cells. Adenosine modulates the tumor microenvironment by interacting with the high affinity ADA-2 adenosine receptor (A2AR) on natural killer cells, limiting their mobility and cytotoxic potential. It is known that A2AR plays an important role in T-cell activation, proliferation, and expansion alongside the CTLA-4 receptor. Therefore, A2AR blockage would appear to be a promising anticancer strategy and A2AR and CD73 inhibitors are now entering early-stage clinical trials in multiple malignancies including non-small cell lung cancer (NSCLC), melanoma and TNBC (NCT03719326, NCT03616886) [9]. Furthermore, another subtype of adenosine receptor A2B (A2BR), which is also stimulated by hypoxia-induced factor-1, plays a critical role in breast cancer stem-cell specification (BCSC). This mechanism is crucial, as proliferation and expansion of myeloid-derived suppressor cells is promoted by A2BR activation, which negatively impacts T-cell mediated tumor activity. The BCSC enrichment is promoted by the adenosine-A2BR complex, which activates kinase C-δ and leads to phosphorylation of the signal transducer and activator of transcription—STAT3. As a result, increased production of two vital mediators of the BCSC phenotype, i.e., IL-6 and pluripotency factor NANOG (a pluripotency homeobox master molecule), significantly increases. TNBC belongs to a subgroup of cancers, among which a significantly elevated concentration of A2BR mRNA is observed, compared to other BC subtypes. Therefore, this new immunomodulatory strategy has the potential as a new therapeutic option for TNBC [10].

Recent studies suggest that bevacizumab, known as Avastin, an antibody targeting vascular endothelial growth factor-A (VEGFR-A), apart from its well-known inhibitory effect in angiogenesis, may also have immunomodulatory potential. Changing the tumor immune microenvironment from immunosuppressive to immunopermissive by bevacizumab includes the conversion of macrophages into APCs, which enhances ADCC. It also increases vascular permeability and thus enables immune checkpoint inhibitors closer access to the tumor microenvironment [11]. In addition, VEGF increases hypoxia, which is one of the triggers for adenosine secretion. Adding an antiangiogenic drug to immunotherapy has been proven successful in hepatocellular carcinoma (HCC) or advanced NSCLC. According to the IMpower110 study, NSCLC patients with high PDL-1 expression may benefit from bevacizumab + atezolizumab therapy, as anti-VEGF seems to enhance the efficacy of anti-PDL-1 treatment [12].

Another immune candidate worthy of attention is the bispecific antibody (bsAb), which is a type of protein that simultaneously targets two different antigens. For TNBC treatment, most bsAbs target the CD3 + antigen on T-cells and various other antigens on tumor cells such as mesothelin, P-cadherin, carcinoembryonic antigen-related cell adhesion molecule 5 (CEACAM 5), and epithelial cell adhesion molecule (epCAM). A special subtype of bsAb- bispecific T-cell engager (BiTe) can be defined as an artificial bispecific monoclonal antibody consisting of two single-chain variable fragments (scFv), one of which connects to T-cells through the CD3 receptor and the other to a tumor-specific antigen [13].

One of the potential targets in the treatment of TNBC is Mucin 1 (MUC1) also known as EMA or CD227, a glycoprotein belonging to a large group of proteins overexpressed on mammillary cells. Their role is to produce mucous, creating a physical barrier and therefore facilitating increased antiviral protection. Moreover, mucins engage several strategies to avoid host immunity, including (1) reducing the number of TILs in the tumor microenvironment, (2) modulating immune cell signaling via co-stimulatory or co-inhibitory molecules, and (3) stimulating proinflammatory cytokine production by binding promoter regions of interleukin-6 (IL-6) and tumor necrosis factor-alpha (TNF-alpha). The CD3 xMUC1 bsAb activates cytokine-induced killer cells and it can recruit CD3 and CD28 cytotoxic T-cells into the microenvironment of a tumor overexpressing MUC-1 antigens [14]. Other BiTe currently being evaluated in xenograft mouse models include CD3 x EGFR Bite, which induces cytotoxic activity against EGFR-expressing TNBC cells [15].

**Fig. 1:**
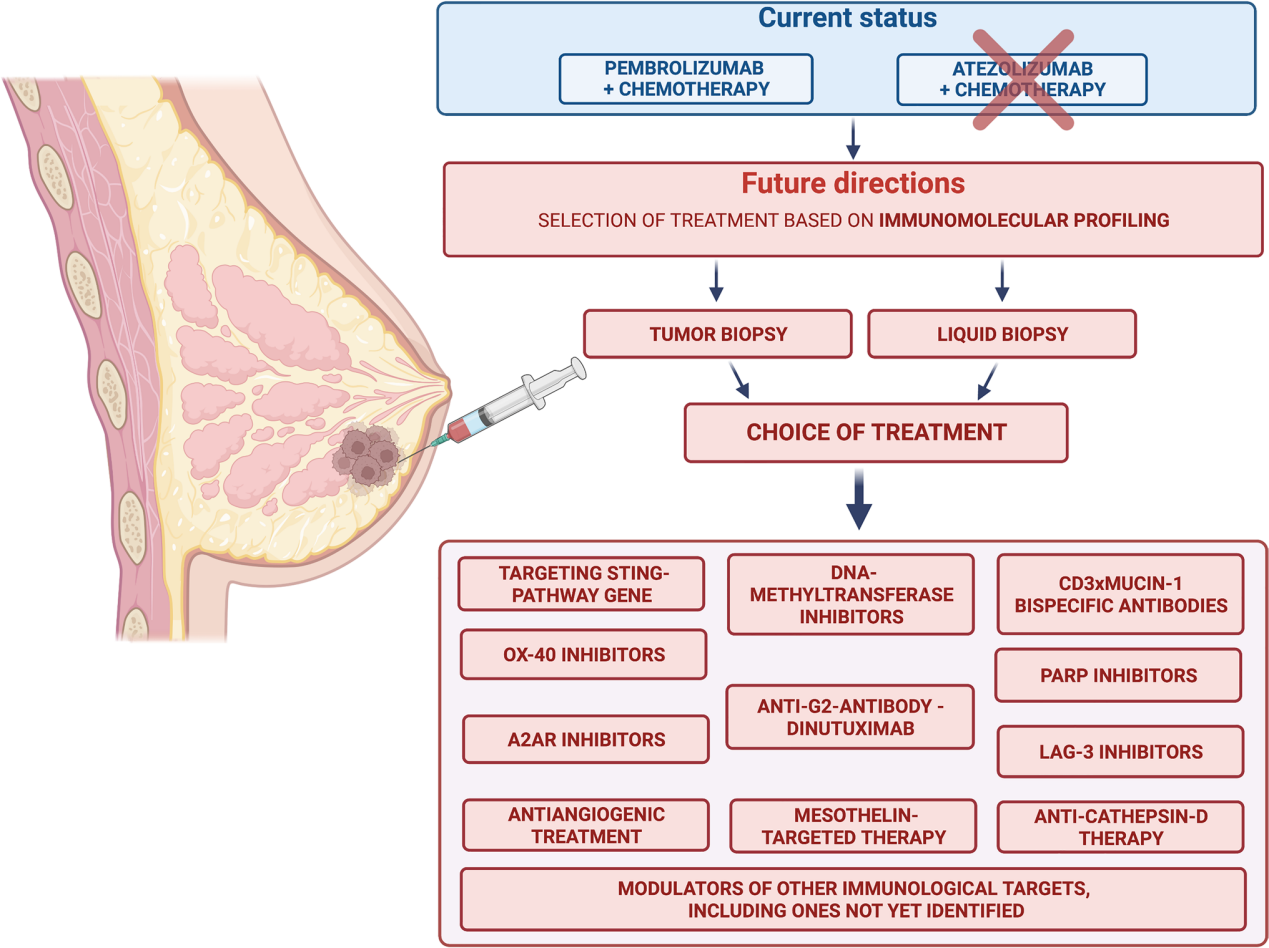
Status and future perspectives of immunotherapy for triple-negative breast cancer patients. Created with BioRender.com accessed on 5.8.2022

Recently the development of viral genetic engineering creates a new pathway for the delivery of pro-apoptotic genes or agents directly into cancer cells. As of now, results are limited, but cell vaccines, primarily based on the oncolytic vesicular stomatitis virus, may improve the prognosis of TNBC patients by stimulation of CD8 + and NK cells [16]. Finally, another strategy is to increase the production of the main anti-cancer cytokine, interleukin-12 (IL-12) using the oncolytic herpes simplex virus, which can selectively induce apoptosis of cancer cells using multiway activation of the immunological system. Immunoviral therapy has the potential to become a promising method in breast cancer immunotherapy [13]. Figure 1 presents a summary of the emerging options for immunotherapy in patients with TNBC as described in the above discussion.

## Conclusion

Despite the initial disappointment, following the withdrawal of accelerated approval for atezolizumab, multiway research has brought new hope that finding an effective treatment for TNBC will not continue to remain a major unmet need for both patients and clinicians. Novel immunomodulatory strategies, which are now at an early developmental stage, may potentially open a whole new era for breast cancer therapy. Clearly, not all immunological targets which may be relevant in the treatment of patients with TNBC have been identified. Some of the abovementioned immunological targets may not stand up to clinical testing, nor are they likely to be applicable to all patients with TNBC. Future research will be concerned not only with the identification of new immunological targets, but also with the incorporation of immunomolecular profiling into clinical practice to identify those patients with TNBC likely to derive the most benefit from immunotherapy. Therefore, we strongly believe that we are at the end of the beginning, and not the beginning of the end in immunotherapy for triple-negative breast cancer.

## References

[1] Won KA, Spruck C (2020). Triple-negative breast cancer therapy: Current and future perspectives (Review). International Journal of Oncology.

[2] Emens LA (2021). Immunotherapy in triple-negative breast cancer. Cancer Journal.

[3] Miles D, Gligorov J, André F, Cameron D, Schneeweiss A, Barrios C, Xu B, Wardley A, Kaen D, Andrade L, Semiglazov V, Reinisch M, Patel S, Patre M, Morales L, Patel SL, Kaul M, Barata T, O'Shaughnessy J (2021). Primary results from IMpassion131, a double-blind, placebo-controlled, randomised phase III trial of first-line paclitaxel with or without atezolizumab for unresectable locally advanced/metastatic triple-negative breast cancer. Annals of Oncology.

[4] Yadav R, Redmond WL (2022). Current clinical trial landscape of OX40 agonists. Current Oncology Reports.

[5] Stovgaard ES, Kümler I, List-Jensen K, Roslind A, Christensen IJ, Høgdall E, Nielsen D, Balslev E (2022). Prognostic and clinicopathologic associations of LAG-3 expression in triple-negative breast cancer. Applied Immunohistochemistry & Molecular Morphology.

[6] Licht JD, Bennett RL (2021). Leveraging epigenetics to enhance the efficacy of immunotherapy. Clinical Epigenetics.

[7] Ashraf Y, Mansouri H, Laurent-Matha V, Alcaraz LB, Roger P, Guiu S, Derocq D, Robin G, Michaud HA, Delpech H, Jarlier M, Pugnière M, Robert B, Puel A, Martin L, Landomiel F, Bourquard T, Achour O, Fruitier-Arnaudin I, Pichard A, Deshayes E, Turtoi A, Poupon A, Simony-Lafontaine J, Boissière-Michot F, Pirot N, Bernex F, Jacot W, du Manoir S, Theillet C, Pouget JP, Navarro-Teulon I, Bonnefoy N, Pèlegrin A, Chardès T, Martineau P, Liaudet-Coopman E (2019). Immunotherapy of triple-negative breast cancer with cathepsin D-targeting antibodies. Journal for Immunotherapy of Cancer.

[8] Ly S, Anand V, El-Dana F, Nguyen K, Cai Y, Cai S, Piwnica-Worms H, Tripathy D, Sahin AA, Andreeff M, Battula VL (2021). Anti-GD2 antibody dinutuximab inhibits triple-negative breast tumor growth by targeting GD2+ breast cancer stem-like cells. Journal for Immunotherapy of Cancer.

[9] Young A, Ngiow SF, Gao Y, Patch AM, Barkauskas DS, Messaoudene M, Lin G, Coudert JD, Stannard KA, Zitvogel L, Degli-Esposti MA, Vivier E, Waddell N, Linden J, Huntington ND, Souza-Fonseca-Guimaraes F, Smyth MJ (2018). A2AR adenosine signaling suppresses natural killer cell maturation in the tumor microenvironment. Cancer Research.

[10] Lan J, Lu H, Samanta D, Salman S, Lu Y, Semenza GL (2018). Hypoxia-inducible factor 1-dependent expression of adenosine receptor 2B promotes breast cancer stem cell enrichment. Proceedings of the National Academy of Sciences of the United States of America.

[11] Chen W, Shen L, Jiang J, Zhang L, Zhang Z, Pan J, Ni C, Chen Z (2021). Antiangiogenic therapy reverses the immunosuppressive breast cancer microenvironment. Biomark Research.

[12] Herbst RS, Giaccone G, de Marinis F, Reinmuth N, Vergnenegre A, Barrios CH, Morise M, Felip E, Andric Z, Geater S, Özgüroğlu M, Zou W, Sandler A, Enquist I, Komatsubara K, Deng Y, Kuriki H, Wen X, McCleland M, Mocci S, Jassem J, Spigel DR (2020). Atezolizumab for first-line treatment of PD-L1-selected patients with NSCLC. New England Journal of Medicine.

[13] Luo C, Wang P, He S, Zhu J, Shi Y, Wang J (2022). Progress and prospect of immunotherapy for triple-negative breast cancer. Frontiers in Oncology.

[14] Lee DH, Choi S, Park Y, Jin HS (2021). Mucin1 and Mucin16: Therapeutic targets for cancer therapy. Pharmaceuticals (Basel).

[15] Stamm H, Oliveira-Ferrer L, Grossjohann EM, Muschhammer J, Thaden V, Brauneck F, Kischel R, Müller V, Bokemeyer C, Fiedler W, Wellbrock J (2019). Targeting the TIGIT-PVR immune checkpoint axis as novel therapeutic option in breast cancer. Oncoimmunology.

[16] Niavarani SR, Lawson C, Boudaud M, Simard C, Tai LH (2020). Oncolytic vesicular stomatitis virus-based cellular vaccine improves triple-negative breast cancer outcome by enhancing natural killer and CD8+ T-cell functionality. Journal for Immunotherapy of Cancer.

